# Aircraft deicing based on large vibration of wings

**DOI:** 10.1371/journal.pone.0308358

**Published:** 2024-09-19

**Authors:** Qian Du, Piao Wang, Dongdong Li

**Affiliations:** 1 College of Aerospace Engineering, Nanjing University of Aeronautics and Astronautics, Nanjing, China; 2 Campus Hospital of NUAA, Nanjing University of Aeronautics and Astronautics, Nanjing, China; COMSATS University Islamabad, PAKISTAN

## Abstract

Since aircraft icing will decrease the ability of aircraft to generate lift, it is significant to consider the aircraft deicing problem. The paper presents an aircraft deicing method based on the cracking of the ice layer caused by the large deformations of wings. To describe the deformation of wings, the absolute coordinate-based formulation is used. The aircraft with high aspect ratio wings is simplified as a hub-beam system. Such a rigid-flexible system with the fast rotation speed of hub and the large deformation of the beam is modeled using absolute coordinate-based formulation accurately. The maneuver of the rigid body will lead to the large deformation of wings to do the de-icing. Numerical examples are presented to reveal that the maximum tensile strength on the wing surface with sinusoidal control torques with some amplitudes and frequencies is larger than the ice’s tensile strength. Hence, the proposed de-icing method based on the aircraft maneuvering is potential.

## 1. Introduction

Aircraft icing may occur when supercooled droplets in the cloud collide and adhere to the aircraft during flight [1]. Ice formation can disrupt the smooth airflow on the lift and control surfaces, then decreasing the ability of aircraft to generate lift [2]. Additionally, uncontrolled shedding of ice accumulated on the surface of an aircraft can seriously damage aircraft components [3]. From an economic perspective, Ice can increase the cost of flight operations [4]. Therefore, the installation of ice protection systems on all critical surfaces, such as wing leading edges, stabilizer leading edges and propellers, is required for aircraft.

Generally speaking, ice protection systems can be divided into two types: de-icing systems and anti-Icing systems [5]. The de-icing system removes ice accumulated on the outer surface periodically. There are various methods for de-icing such as pneumatic boots, electromagnetic expulsion, chemical and thermal techniques for ice removal [6–9]. The anti-icing system prevents the formation of ice or frost for a limited amount of time, and they are activated before icing conditions become apparent in order to prevent ice adhesion to the surface. There are mainly three types of anti-icing systems, i.e., hot-air systems, chemical systems and electrical resistance heating (electro-thermal) systems [10]. There is a need for a low-power, reliable de-icing system. There are several potential solutions for de-icing by deformation. Pneumatic boot system is capable of removing accreted ice from aircraft surfaces, but when operating, may affect the aircraft stall characteristics [11]. Electro-impulse de-icing can create a repulsive force which breaks, debonds and expels ice on the skin surface. Due to low energy requirements, the electro-impulse de-icing system is usually suitable for small-aircraft applications [12, 13]. Several researchers demonstrated a system that can use piezoelectric actuators to generate ultrasonic shear waves to remove ice from aluminum plates [14–16].As one of the mechanical de-icing systems, vibration has the advantages of stability, effectiveness, reliability, and low energy consumption, et al.

High-aspect-ratio wing has been widely used in various aircraft, such as civil air-planes, transport airplanes, long-endurance Unmanned Aerial Vehicles (UAVs) and so on. In recent years, people have become increasingly interested in high altitude long endurance (HALE) aircraft. These aircraft are considered for unmanned reconnaissance missions, long-term surveillance, environmental sensing, and communication relaying, with the advantages of being cheaper, closer to the ground and more flexible. In fact, they can be easily recycled for maintenance as long as necessary, and can be moved to different areas if necessary [17]. Solar-powered unmanned aerial vehicles (SPUAVs), using clean, low-cost, and inexhaustible solar energy, the solar powered high altitude long endurance wings can stay in the air for months or even years, acting as atmospheric satellites [18–20]. The aeroelastic characteristics of highly flexible aircraft are studied by Van Schoor and Von Flotow [21]. The complete aircraft was modelled using several modes of vibration, including rigid-body modes. The linear aeroelastic and flight dynamics analysis results of HALE aircraft are presented by Pendaries [22].

It is worth pointing out that the above studies on de-icing for high-aspect-ratio wings did not consider the geometrical nonlinearities due to large deflections. However, as a result of the high flexibility and large aspect ratios, large deflections may lead to about 25% of wing semi-span. The aircraft with high-aspect-ratio wings is a rigid-flexible system essentially. The large maneuver of the aircraft may result in the large vibrations of high-aspect-ratio wings, which could remove ice on the wings. In the field of multi-body dynamics, an effective method to model the rigid-flexible system with large deformation and fast motion is the absolute coordinate-based formulation [23], in which the rigid and flexible parts are described by absolute coordinate formulation and absolute nodal coordinate formulation, respectively. In absolute coordinate-based formulation, the Cartesian coordinates or the attitude angles of the rigid body is used in the inertial frame. The absolute nodal coordinate formulation was initially proposed by Shabana [24] for the flexible multi-body system with large deformation which has been an important topic and drawn growing attention [25–34]. For example, in Ref. [29], absolute nodal coordinate formulation on the basis of radial point interpolation method is used to build the dynamic model of curved beams and the transient response is analyzed for the curved beams with a steady state angular speed. Ref. [32] derived the dynamics model of soft tissues using the absolute nodal coordinate formulation. In Ref. [34], the accurate modeling of the tether of remotely- controlled underwater vehicle is considered based on a new tether cable element utilizing the absolute nodal coordinate formulation. To improve the computational efficacy, Shi et al. developed an adjoint method to compute the standard eigenvalue and eigenvector derivatives for the LST problem [35]. Also, some works have been done about the control, aerodynamic effects and dynamics modeling for aircraft system. For example, Tan et al. [36] studied the new event-triggered sliding mode control for the anti-unwinding reorientation with multiple attitude constraints and external disturbance. The research by Wang et al. [37] on transition models for transonic boundary layers can provide valuable information on the aerodynamic effects of large deformations. Li et al. [38] developed superhydrophobic shape memory composites that can offers a complementary approach to mechanical deicing. Fu et al. [39] provide insights into thermal management for the analysis of heat exchangers for aero-engine cooling using the Wilson plot method which is relevant to the overall deicing strategy. Wang [40] et al. studied the nonlinear dynamic modeling of rotor systems, which can help improve the modeling accuracy of the hub-beam system.

As far as we know, this is an attempt to present how to clear the ice on the wings by aircraft’s maneuverings which are described by the absolute coordinate-based formulation. The organization of the paper is as follows. In Section 2, the aircraft with high-aspect-ratio wings is simplified as a hub-beam system, and described by absolute coordinate-based formulation. Then, a lot of case studies are presented to reveal the maximum tensile strength on the wing surface with sinusoidal control torques with various amplitudes and frequencies in Section 3. The conclusions are drawn in Section 4.

## 2. Dynamic model

**Fig 1:**
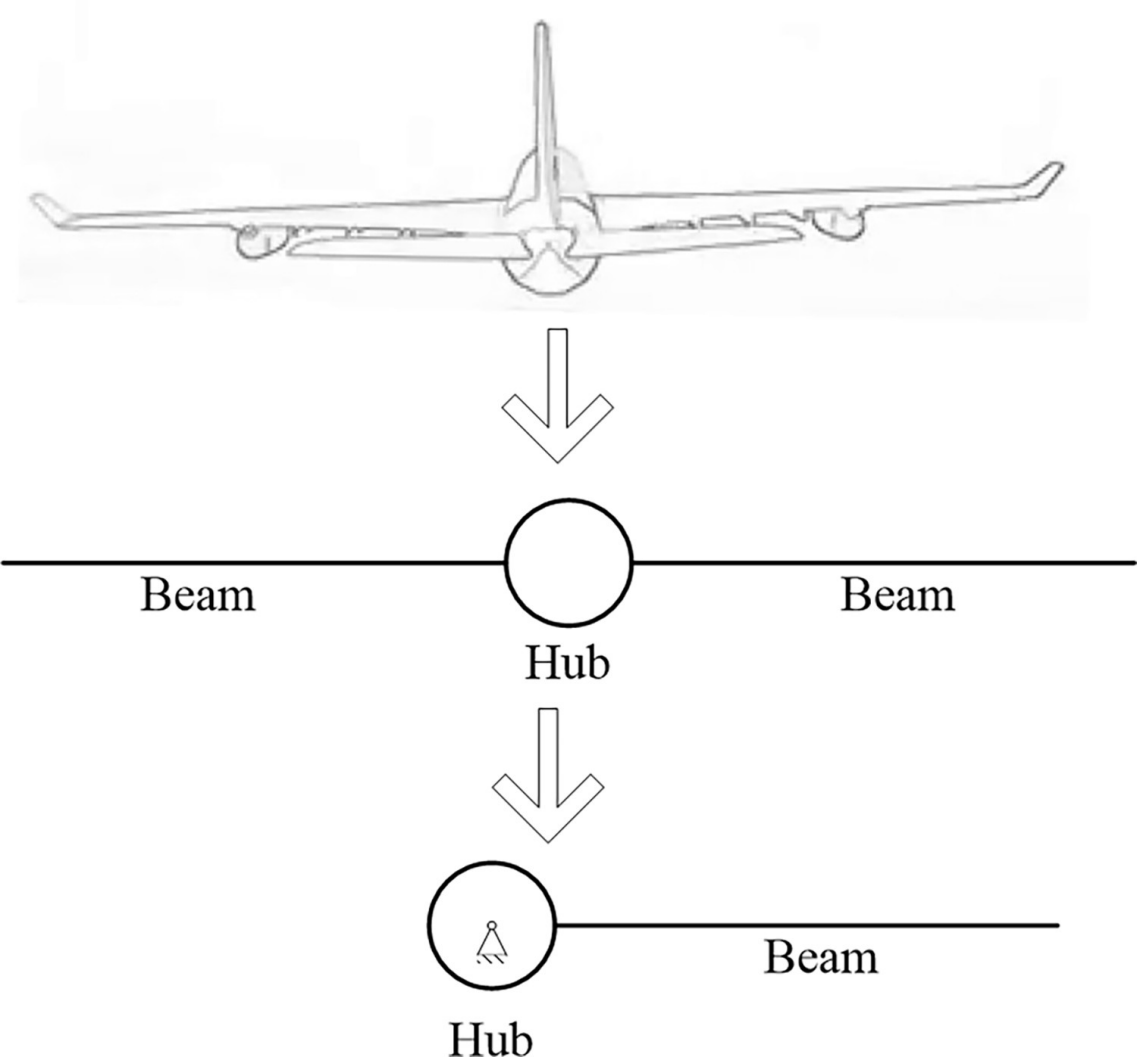


As shown in Fig 1, the aircraft with long wings is simplified as a planar hub-beam system and only half structure is considered due to the symmetry of two beams. Furthermore, as well known, the vibration of the beam is mainly coupled with the rotation of the hub. Hence, the aircraft system is treated as a rotating hub-beam system shown in the last subgraph of Fig 1.

The radius of the hub is represented by *R*. The length of the homogeneous and isotropic beam is l, which is so long that the shear effect in the beam can be neglected. In the case of fast movement of hub and large deformation of the beam, the absolute coordinate-based formulation can describe the motion of the hub-beam system accurately. In absolute coordinate-based formulation, all the coordinates all defined in an inertial frame; hence, coordinate transformation is not required and the mass matrix is constant. In what follows, the hub and the beam are modeled, and then the constraint conditions depict the connection between the hub and the beam.

Firstly, an absolute attitude angle *θ* is used to describe the rotation of the hub. The dynamic equation of the hub reads

$$J_{r}\ddot{\theta }=\tau$$ (1)

where *J*_*r*_ is the rotary inertia of the hub and *τ* is the control torque acting on the hub.

Secondly, the 2D slender beam element is adopted to model the beam via the so-called absolute nodal coordinate formulation (ANCF). The beam is divided into *n* elements with the same length *l*_*e*_ = *l*/*n*. The displacement field of an arbitrary element can be expressed in the global coordinate system as

$$r={\left[\begin{array}{cc} X & Y \end{array}\right]}^{T}=Se$$ (2)

where X and Y represent the nodal coordinates in the global coordinate system, respectively. ***S*** is the shape function matrix and ***e*** is the vector of element nodal coordinates

$$e={\left[\begin{array}{cccccccc} r_{i1} & r_{i2} & \frac{\partial r_{i1}}{\partial x} & \frac{\partial r_{i2}}{\partial x} & r_{k1} & r_{k2} & \frac{\partial r_{k1}}{\partial x} & \frac{\partial r_{k2}}{\partial x} \end{array}\right]}^{T}$$ (3)

$$\begin{array}{l} S=[S_{1}I\;S_{2}I\;S_{3}I\;S_{4}I],\\ S_{1}=1-3\xi^{2}+2\xi^{3},\;S_{2}=l(\xi -2\xi^{2}+\xi^{3}),\\ S_{3}=3\xi^{2}-2\xi^{3},\;S_{4}=l(\xi^{3}-\xi^{2}),\;\xi =x/l_{e} \end{array}$$ (4)

in which, *x* is the coordinate of an arbitrary point on the beam element in the undeformed configuration. The vector ***e*** includes the global displacements *r*_*i*1_, *r*_*i*2_, *r*_*k*1_, *r*_*k*2_ and the global slopes of the element nodes $\frac{\partial r_{i1}}{\partial x}$, $\frac{\partial r_{i2}}{\partial x}$, $\frac{\partial r_{k1}}{\partial x}$, $\frac{\partial r_{k2}}{\partial x}$.

The kinetic energy of a beam element yields

$$T=\frac{1}{2}\int_{0}^{l_{e}}\rho {\dot{r}}^{T}\dot{r}dx=\frac{1}{2}{\dot{e}}^{T}M^{e}\dot{e}$$ (5)

where *ρ* represents the linear density of the beam. The mass matrix of a beam element reads

$$M^{e}=\int_{0}^{1}\rho S^{T}Sl_{e}d\xi$$ (6)

which is a constant matrix.

The configuration of the beam element at time t can be defined as ***r*** = ***r***(*x*), 0≤*x*≤*l*. Both axis and bending effect should be considered in order to express the strain energy. The longitudinal strain *ε*_*l*_ can be defined as

$$\varepsilon_{l}=\frac{1}{2}({r^{\prime }}^{T}r^{\prime }-1)$$ (7)

and the bending deformation is characterized by the curvature *κ* of the central line of the cross-section of the beam as following

$$\kappa =\frac{\left|r^{\prime }\times r^{\prime \prime }\right|}{{\left|r^{\prime }\right|}^{3}}$$ (8)

where $r^{\prime }=\frac{dr}{dx}$. The strain energy is given by

$$U=U_{l}+U_{t}=\frac{1}{2}\int_{0}^{l}[EA\varepsilon_{l}^{2}+EI\kappa^{2}]dx$$ (9)

The vector $Q_{k}^{e}$ of the elastic forces is defined as

$$Q_{k}^{e}={\left(\frac{\partial U}{\partial e}\right)}^{T}$$ (10)

The vector $Q_{e}^{e}$ of the external forces is defined by using the virtual work and set as zero vector in this study.

By using the principle of virtual work, one can obtain the following dynamic equations of a finite element in a matrix form as

$$M^{e}\ddot{e}+Q_{k}^{e}=Q_{e}^{e}$$ (11)

The dynamics equations of the whole beam can be assembled using Eq (11) as

$$M_{b}\ddot{e}+Q_{k}^{b}=Q_{e}^{b}$$ (12)

Thirdly, the dynamic equations of the hub-beam system can be established, by combining Eq (12) and Eq (1), as

$$M\ddot{q}+Q_{k}=Q_{e}$$ (13)

where ***q***, ***M***, ***Q***_*k*_ and ***Q***_*e*_ are the generalized coordinates vector, the whole mass matrix, the whole elastic forces vector and the whole external force vector, respectively. The detailed expressions of the four symbols are

$$q={\left[\begin{array}{cccc} q_{1} & q_{2} & \ldots & q_{4(n+1)+3} \end{array}\right]}^{T}={\left[\begin{array}{cccccccc} X_{o} & Y_{o} & \theta & e_{1} & e_{2} & e_{3} & \ldots & e_{4(n+1)} \end{array}\right]}^{T}$$ (14)

$$M=[\begin{array}{cc} M_{r} & 0\\ 0 & M_{b} \end{array}],\;Q_{k}=[\begin{array}{c} 0\\ Q_{k}^{b} \end{array}],\;Q_{e}=[\begin{array}{c} Q_{r}\\ Q_{e}^{b} \end{array}]$$ (15)

As the fourth step, the constraint equations should be taken into account to describe the hub-beam system properly. That is, one end of the beam should be attached on the hub rigidly. The expressions of the constraint yield

$$\left\{ \begin{array}{l} R\;\cos\;q_{1}-q_{2}=0\\ R\;\sin\;q_{1}-q_{3}=0\\ \cos\;q_{1}-q_{4}=0\\ \sin\;q_{2}-q_{5}=0 \end{array}\right.$$ (16)

Eq (16) can be paraphrased into the matrix form as

Φ(q)=0 (17)

Therefore, the complete dynamic equations of the hub-beam system in the matrix form yields

$$\left\{ \begin{array}{l} M\ddot{q}+Q_{k}=Q_{e}\\ \Phi (q)=0 \end{array}\right.$$ (18)

In the study, the general-*α* method is used to solve Eq (18) numerically, which is a set of differential algebraic equations.

## 3. Numerical analysis

### 3.1. System analysis based on FFT

**Fig 2:**
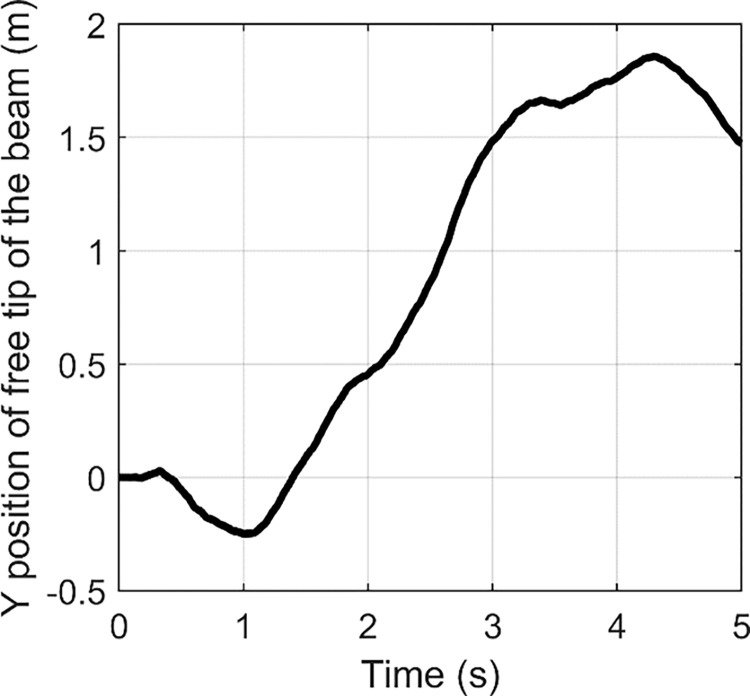


**Fig 3:**
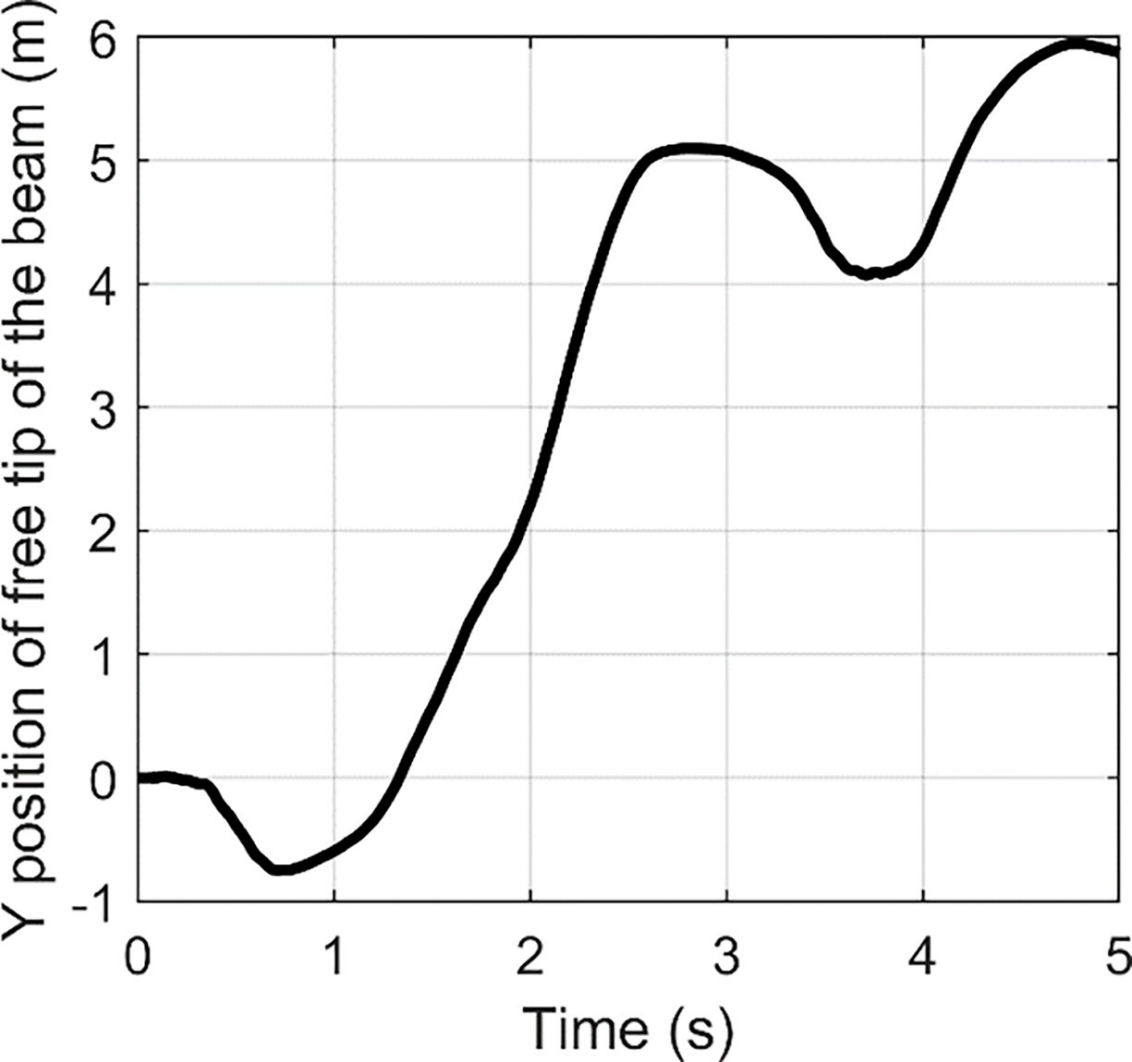


**Fig 4:**
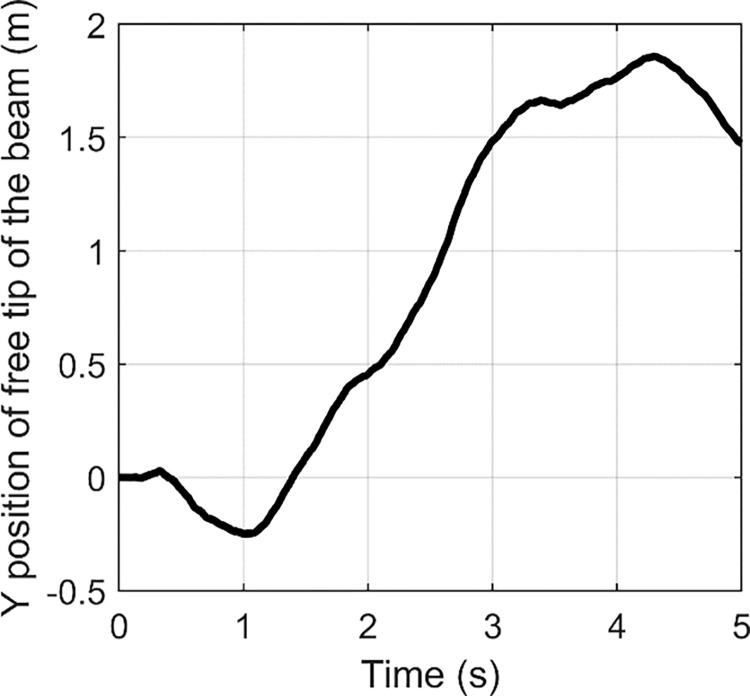


In order to obtain larger tensile strength, a fast Fourier transform (FFT) is usually used to identify the main vibration frequency of the structure of concern with an impulse input for linear system. FFT is an algorithm which computes the discrete Fourier transform of a sequence. Fourier analysis can convert a signal from time domain to frequency domain. It is very useful for analysis of time-dependent phenomena. An FFT can rapidly calculate such transformations by factorizing the DFT matrix into a product of sparse factors [41]. Essentially, FFT can be used to assess the frequency distribution of the flexible vibration. The time histories of the free tip of wing under three impulse inputs are shown in Figs 2–4.

**Fig 5:**
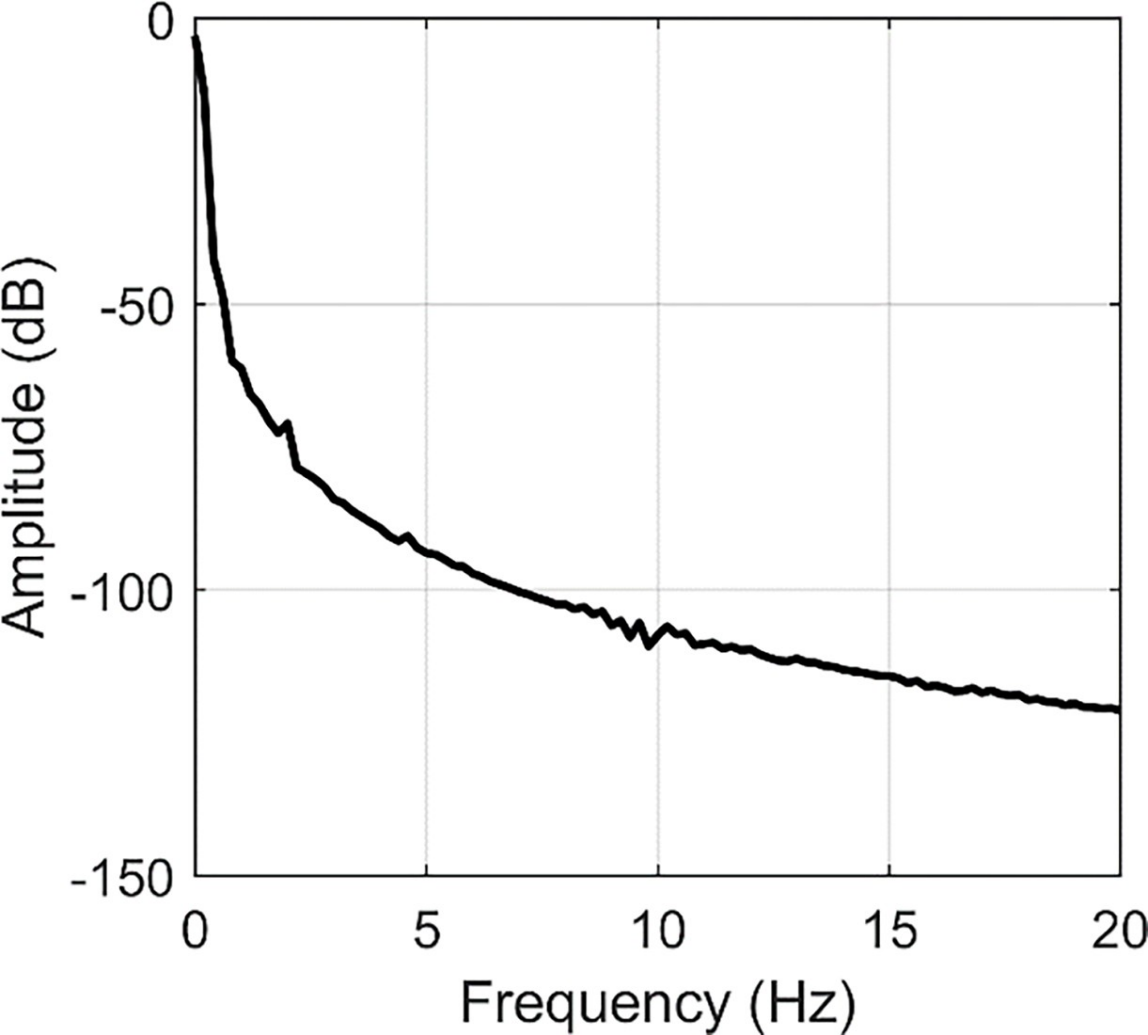


**Fig 6:**
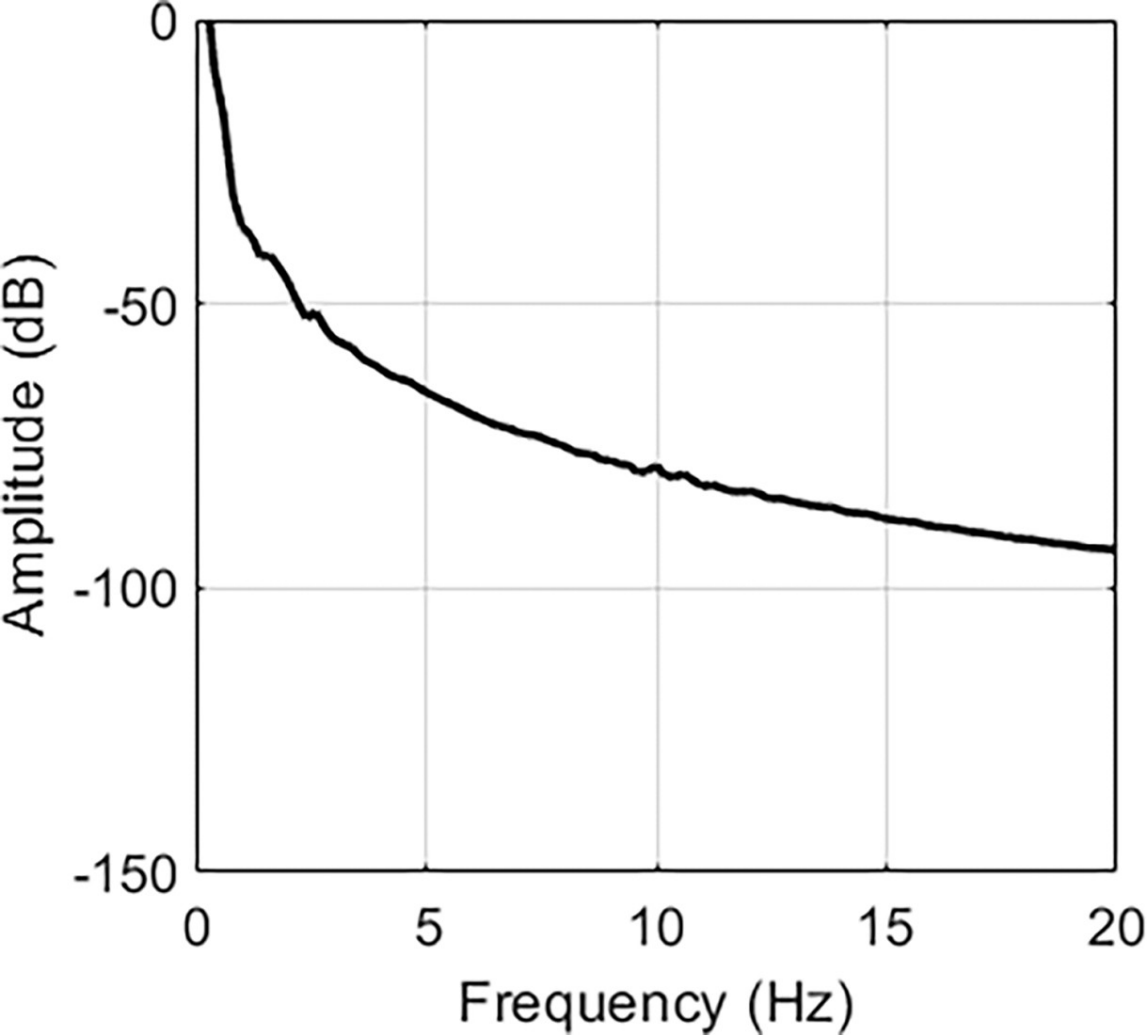


**Fig 7:**
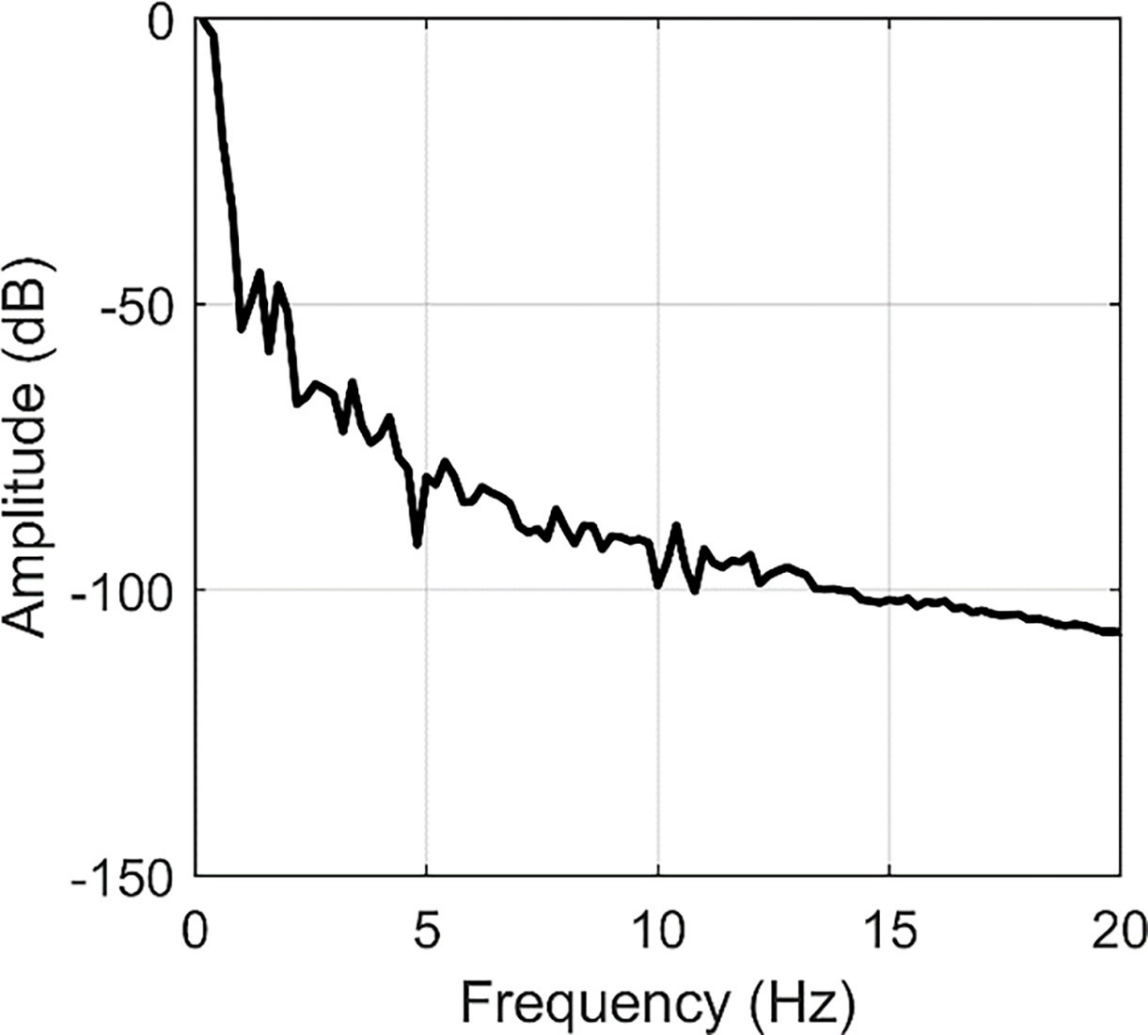


Based on FFT, the frequency responses are indicated in Figs 5–7. From Fig 5, we can find two peaks at 2 Hz and 4.6 Hz. In Fig 6, two peaks are located at 2.6 and 10 Hz. Fig 7 contains peaks at 1.4 Hz, 1.8 Hz, 3.4 Hz, 4.2 Hz, 5.4 Hz, 7.8 Hz and 10.4 Hz. Obviously, the main vibration frequency is not a constant. The essential reason is that the system is under large deformation, hence, it is nonlinear. Therefore, it is not easy to analyse the system response based on the traditional linear methods and in the next section, numerical examples are presented with various amplitudes and frequencies.

### 3.2. Numerical analysis with various amplitudes and frequencies

The aim of this section is to study the maximum tensile strength on the wing surface with sinusoidal control torques with various amplitudes and frequencies via some numerical examples. The mass and radius of the hub are selected as 5 kg and 1 m. The linear density, length, thickness, width and Young’s modulus of the beam is 0.2 kg/m, 5 m, 2 mm, 0.9222 m and 7 GPa, respectively. The control torque is expressed as

$$\tau =\tau_{0}\;\sin(\omega_{0}t)$$ (19)

**Fig 8:**
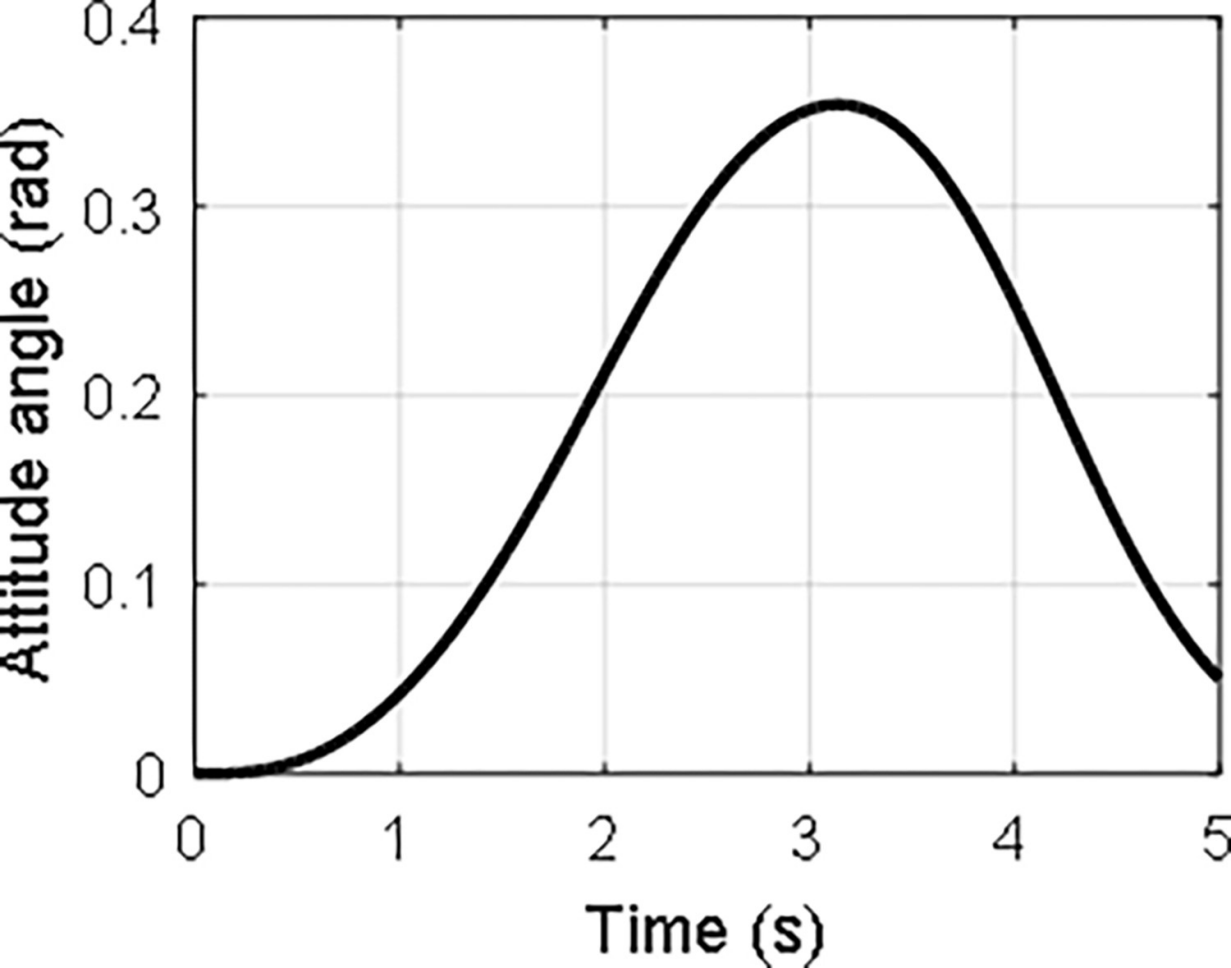


**Fig 9:**
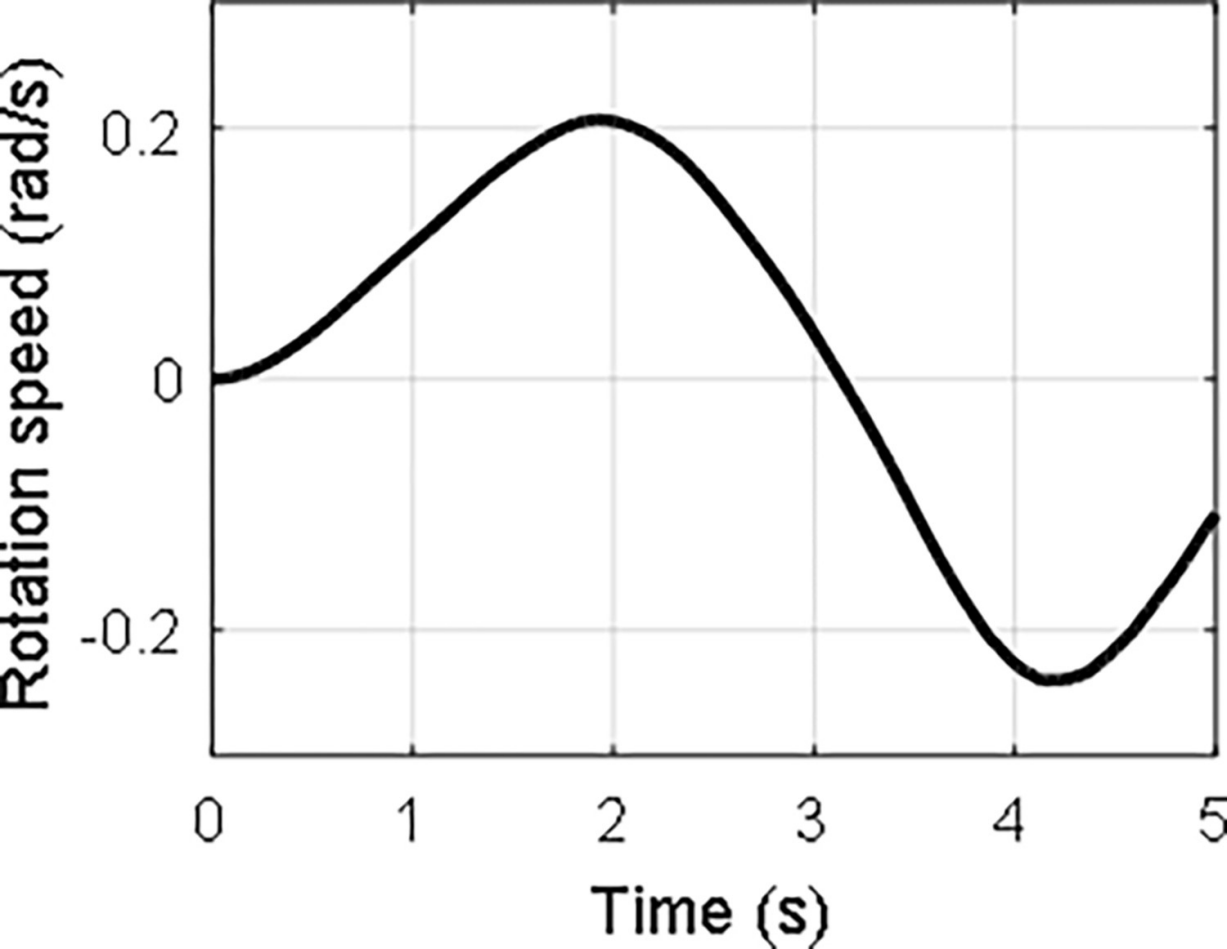


**Fig 10:**
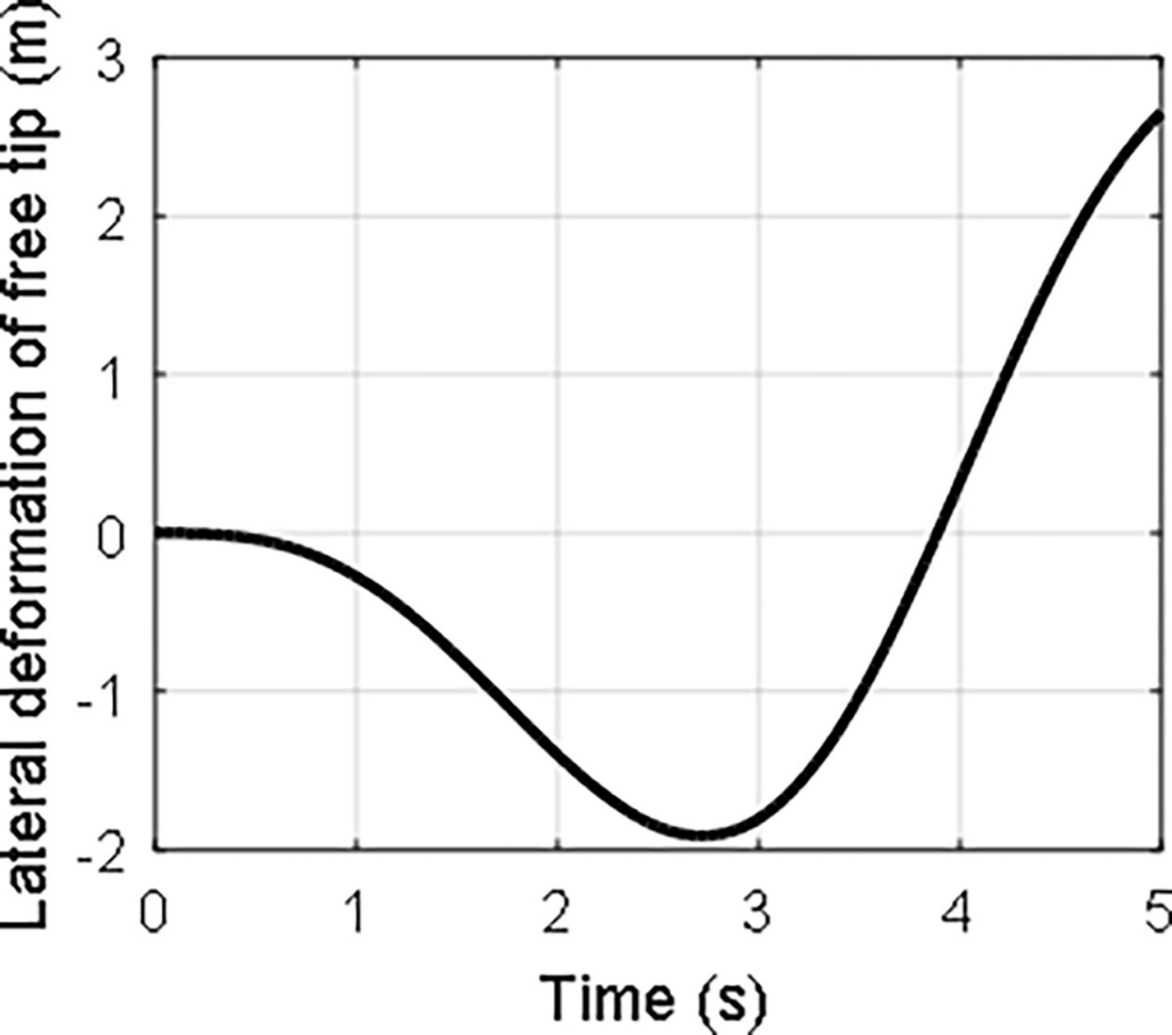


In the case that *τ*_0_ = 1 and *ω*_0_ = 1, the time history of the attitude angle and the rotation speed of the hub, and the lateral deformation of the beam are shown in Figs 8–10, respectively. It can be seen that the hub-beam system is subject to fast rotation speed and large deformation and the absolute coordinate-based formulation can evaluate the motion of the hub-beam system accurately.

**Fig 11:**
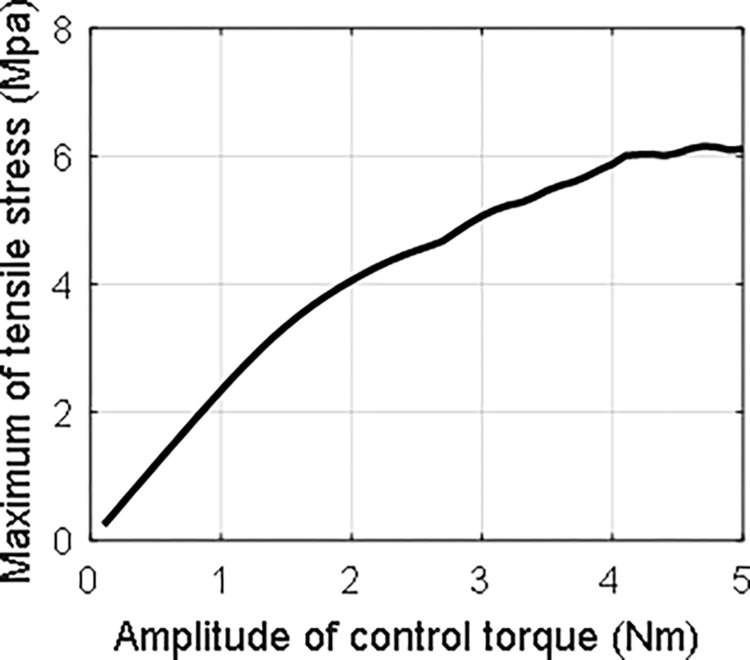


The maximum tensile strength on the wing surface in this case within 5 seconds is about 2.3 MPa. To investigate the maximum tensile strength on the wing surface with different control input amplitude, *τ*_0_ is set from 0.1 to 5 with *ω*_0_ being one. The step is chosen as 0.1. The result is shown in Fig 11. Obviously, the maximum tensile strength almost increases gradually with the enlargement of the amplitude of control torque.

**Fig 12:**
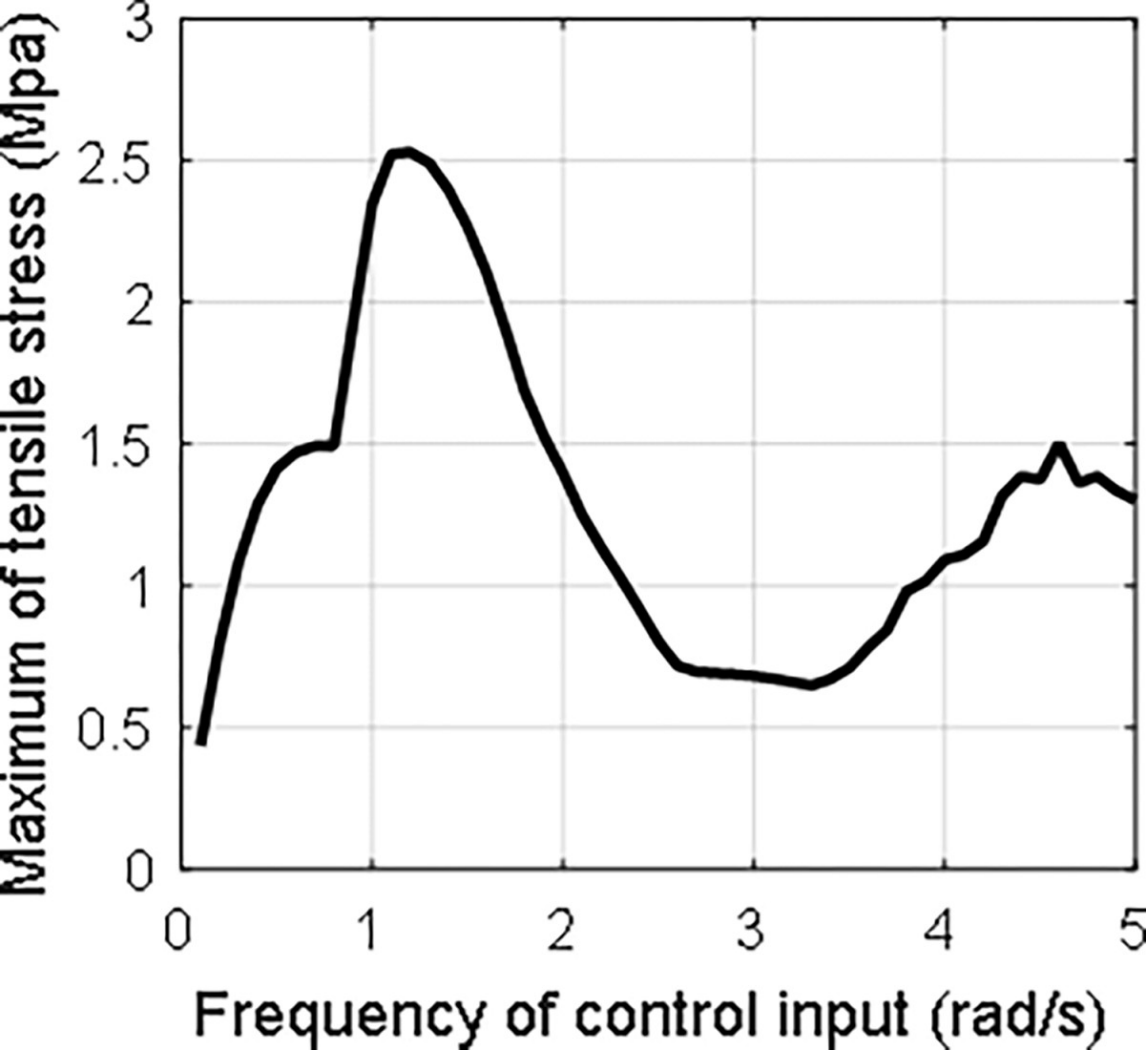


Then, *τ*_0_ is chosen as 1 and *ω*_0_ is set from 0.1 to 5 with the step being 0.1. The variation of the maximum tensile strength on the wing surface with different control input frequency is given in Fig 12. It can be seen that the two extreme points occur at about 1.2 rad/s and 4.6 rad/s.

In traditional vibration theory, the natural frequency of a cantilever beam subject to small deformation can be calculated by the following formula.

$$\omega_{n}={\left(s_{n}l\right)}^{2}\sqrt{\frac{EI}{\rho l^{4}}},\;n=1,2,3\ldots$$ (20)

where *s*_1_*l* = 1.8751 and *s*_2_*l* = 4.6941. Hence, the first and second order natural frequency of the beam with the parameters shown at the beginning of this section are 0.6290 rad/s and 3.9417 rad/s.

Apparently, the extreme points do not locate at the first and second order natural frequency. The main reason may be that the large vibration of the beam cannot be described using traditional vibration theory.

The tensile strength of ice usually varies from 0.7–3.1 MPa. Hence, it is obvious that the ice can be removed by the large vibration of wing resulting from the large rotation of the aircraft.

## 4. Conclusions

This study focuses on the feasibility of aircraft de-icing for those with elongated wings, modeled as a dynamic rotating hub-beam system. The investigation reveals that the rigid-flexible coupling inherent in the system’s design can induce wing vibrations, which can effectively remove ice. The absolute coordinate-based formulation is employed to accurately depict the system’s rapid movements and large deformations. Some numerical simulations show that the maximum tensile strength exerted on the wing surface under specific control torques surpasses the ice’s tensile strength, validating the potential of aircraft maneuvering for de-icing. In the future, a dynamics model of the wing structures with more details and some experimental tests can be built to verify such a de-icing concept.

## Supporting information

S1 File MATLAB program for numerical analysis. (ZIP)
